# Effect of Breed on the Volatile Compound Precursors and Odor Profile Attributes of Lamb Meat

**DOI:** 10.3390/foods9091178

**Published:** 2020-08-26

**Authors:** Can Zhang, Hao Zhang, Ming Liu, Xin’gang Zhao, Hailing Luo

**Affiliations:** 1 State Key Laboratory of Animal Nutrition, College of Animal Science and Technology, China Agricultural University, Beijing 100193, China; zhang_can@cau.edu.cn (C.Z.); liu-ming@cau.edu.cn (M.L.); 1404010216@cau.edu.cn (X.Z.); 2 College of Food Science and Nutritional Engineering, China Agricultural University, Beijing 100083, China; zhanghaocau@foxmail.com

**Keywords:** lamb, breed, fatty acid, amino acid, volatile compounds

## Abstract

The objective was to characterize the effect of breed on the volatile compound precursors and odor profile attributes and to provide an insight into improving the lamb production and meat flavor. Three-month-old Tan (*n* = 10), Hu (*n* = 10) and Dorper lambs (*n* = 10) were raised for 90 days in single barns. *Longissimus thoracis et lumborum* muscle of all lambs were collected for analysis of intramuscular fat, fatty acids, amino acids, and volatile compounds. The results showed Tan and Hu accumulated more intramuscular fat and saturated fatty acid than Dorper. However, Tan had lower linoleic acid, alpha linolenic acid and total polyunsaturated fatty acid proportion than Dorper. Amino acid in Dorper was significantly higher than Tan and Hu. Furthermore, (*E*)-2-hexenal was only found in Tan lambs, while (*E*)-2-nonenal and (*E*,*E*)-2,4-nonadienal were only found in Dorper lambs. Hu had the fewest volatile compounds. The results of this study demonstrated that Dorper had larger proportion of polyunsaturated fatty acids (PUFA), amino acid and volatile compounds than Tan and Hu. However, the specific PUFA derivates of Dorper had a negative impact on the odor profile. Hence, we suggest that further works should be focused on crossbreed lambs by Dorper and Tan, to enhance the lamb production and improve meat flavor.

## 1. Introduction

The consumer acceptability of lamb meat can be influenced by many attributes, such as meat nutritional quality and sensory profile [1,2]. The sensory acceptability of lamb meat also depends on regional or cultural factors, and varies across countries [3,4]. Therefore, more and more researches focused on improving meat flavor, sensory and odor profile, to meet the increasing need of consumers [5,6]. Some studies worked on different pasture type [7] or supplementations [8], and others concentrated on animal pre- and post-slaughter factors [9,10], to discuss the available ways of acquiring the ideal mutton flavor. Beyond that, animal genotype is one of the dominating factors [11]. Volatile compounds and precursors, such as polyunsaturated fatty acid and amino acid, can be different across lamb breeds [12].

Tan is a local fat-tail sheep that is used to produce meat and lambskin. Dorper sheep is a productive breed for meat. Hu sheep is the most common breed for multifetation. The three breeds are generally used to improve lamb production or reproduction. The designated lamb meat for G20 Summit, Tan lamb, of lower “off-flavor” and “mutton smell”, is famous for its great eating quality and pleasant aroma. Our previous studies found the production performance and meat quality of Tan lamb could be affected by grazing time [13,14]. However, the precursors and odor-active compound profile of Tan lamb are not reported. Aldehydes are the basic aroma-active compounds mainly derived from polyunsaturated fatty acids. Some aldehydes have a lower odor threshold so that can be the critical composition for meat odor profile [15]. Furthermore, the oleic acid, linoleic acid, and alpha linolenic acid are the most important aroma-active compound precursors and are likely to be influenced by genotype [16,17]. Besides, the polyunsaturated fatty acids in muscle also affect the different oxidation-derived volatiles in lambs [11]. So, genotype is a basic factor on lamb lipid metabolism and chemical compound synthesis.

Several studies identified some factors influencing meat volatiles or flavor precursors of lamb meat [9,16]. Recent studies also discussed the different volatile profile among species [18,19]. It has been reported that fat-tailed lamb presents a favorable fatty acid profiles, but a worse eating quality [20] than tailed lamb, and the differences in the flavour of lamb meat may be a reflection of differences in the composition of fat depots [21]. Therefore, the objective of this study was to characterize the effect of breed (typical fat-tailed lamb or short-tailed lamb) on the volatile compound precursors and odor profile attributes, and to provide an insight into improving lamb production and meat flavor.

## 2. Materials and Methods 

The experimental animal protocol was admitted by the China Agricultural University Animal Care and Use Committee, Beijing, China.

### 2.1. Animal Management and Sample Collection

The experiment started in August 2018, with a total of 30 male lambs from 3 breeds; fat-tailed Tan (*n* = 10), fat-tailed Hu (*n* = 10) and short-tailed Dorper (*n* = 10). Lambs were 3 months old and weighed 20.67 kg for Tan, 19.20 for Hu, and 37.90 kg for Dorper at the beginning of the experiment (published data). Tan, Hu and Dorper lambs were randomly selected from native herds at the NingXia province of China. Lambs inherited the lineage from a same sire within breed and were reared with their dams in feedlots. The dominant forage for lambs in this trial prior to weaning included maize straw and alfalfa hay, which is considered typical for three breeds. All lambs were reared by suckling milk from their dams and were weaned at 3 months of age. After weaning, lambs were raised indoors in single barns for 90 days, and fed with same pellet diet (Table 1), plus mixed hay (40% Alfalfa hay, 40% Caragana hay and 20% Maize straw) at YanChi county (Longitude 107° E, latitude 37° N). All the animals were under unified management during the experimental period.

At the end of the experiment (90 days), all lambs were transported to an abattoir located 60 km from the experimental site, and were slaughtered after 12 h of fasting with access to water. The average live weight of lambs prior to slaughter was 37.15 kg, 35.46 kg and 60.54 kg for Tan, Hu and Dorper, respectively. Each carcass was split along the middle line and stored at 4 °C for 12 h. *Longissimus thoracis et lumborum* (*LTL*) muscle from the fourth to the sixth lumbar vertebrae was removed from both half carcasses. Raw meat samples were trimmed of external fat and divided into slices, then conserved at −20 °C in vacuum packages for subsequent chemical analysis. The LTL from the left side was used for detection of volatile compounds. The other ones were for nutrients analysis.

Limited by the experimental conditions, there was 1 sire of each breed chosen to provide frozen semen in our study. For further studies, it is better to utilize more sires and progenies to provide sufficient genetic coverage, and to be more representative of the breed genotype. 

### 2.2. Chemical Analysis

Total percentages of protein, ether extract (as intramuscular fat, IMF), ash, and moisture were determined according to the Association for Official and Analytical Chemists (AOAC) [22] recommendations.

### 2.3. Fatty Acid Analysis

Briefly, intramuscular fat was extracted using a chloroform/methanol mixture. Gas chromatography was applied for determination of the fatty acid composition. Moreover, 0.3 mg of the extracted fat was weighed in a test tube, and then 5 mL of methanol–NaOH (2%) was added. It was then cooled to room temperature after heating at 80 °C for 2 h. Then, 2.175 mL of 20% BF_3_ was added to the sample, and immersed back into a boiling water bath for 3 min. After that, 1 mL hexane was added to the system and shaken. After 5 min, the system was in three phases, and 0.2 mL of the upper solution was removed and injected into the gas chromatograph apparatus [20]. The chromatographic model: Agilent HP6890, column db-23, was heated at 220 °C for 10 min, then increased to 250 °C at 10 °C/min for 5 min. The detector temperature was 270 °C, the shunt ratio was 20:1, and the column inlet temperature was 250 °C. Fatty acid content was calculated by chromatographic peak area.

### 2.4. Amino Acid Analysis

An amino acid analyzer (L8900, HITACHI, Tokyo, Japan) was applied for amino acids composition according to the AOAC [22]. Then, 0.5 g dried meat samples were weighed accurately and then hydrolyzed by 6 mol/L hydrochloric acid for 24 h at 110 °C in a vacuum environment. After cooling, solution was constant volume, filtered and evaporated. Lithium hydroxide solution (4 mol/L) was used for the alkaline hydrolysis of tryptophan in samples.

The analysis of sulfur-containing amino acids (cysteine, methionine): 0.3 g dried samples were weighed accurately and put into rotatory evaporator bottle, cooled down with water bath for 30 min. Then, 2 mL formic acid was added and placed into the react system under a 0 °C environment for 16 h. Furthermore, 0.3 mL hydrobromic acid (48%) was used as terminator. The system was set into 0 °C for 30 min after shaking, and then dried in a rotatory evaporator. Moreover, 25 mL 6 mol/L hydrochloric acid was then added and hydrolyzed for 24 h. Then, we took ~1 mL filtrate and evaporated it, adding ~3 mL sodium citrate buffer solution and centrifuging the supernatant for analysis.

### 2.5. Volatile Compounds Analysis

#### 2.5.1. Volatile Compound Extraction

According to Machiels [23], meat was thawed naturally at 4 °C, removed fascia and minced. Furthermore, 10 g of sample was put into a 40 mL headspace bottle, then 2 g of sodium chloride solid was added and heated at 120 °C for 30 min after sealing. Then, the whole thing was cooled to room temperature. The system was taken in a water bath at 60 °C and SPME was absorbed for 30 min. The whole absorption process was in a sealing system. 

#### 2.5.2. Volatile Compound Analysis

A gas chromatograph-mass spectrograph (GC-MS, 7890B-5977B, Agilent, Palo Alto, CA, USA) was applied for volatile compound identification [19]. GC separation was carried out on a DB-5 nonpolar column (60 m × 0.32 mm I.D., 1 μm film thickness, J & W Scientific, Rancho Cordova, CA, USA). The temperature program was 3 min at 40 °C, with a ramp of 6 °C /min to 230 °C, and it was held for 3 min. Helium was used as the carrier gas, resulting in a flow of 0.8 mL·min^−1^ at 40 °C.

The mass spectrometer operated in electron impact mode, with a source temperature of 230 °C, an ionising voltage of 70 eV and a scan range from m/z 45 to m/z 350, at 2.76 scans/s. Compounds were identified by first comparing their mass spectra with spectra from the Mainlib/NIST/Wiley7 Mass Spectral Database. Volatiles were identified by the comparison of retention indices (RIs) with published RI values. The approximate quantities of the volatiles were estimated by the comparison of their peak areas with that of the n-alkane internal standard obtained from the total ion chromatograms, and the formula of the retention index of the substance, to be tested is as follows:

$$R_{X}=100N+\frac{T_{X}-T_{Y}}{T_{Z}-T_{X}}\times 100$$

*R_x_* is the retention index of the substance; *N* is the carbon number of n-alkanes whose retention time is less than and closest to the substance; *T_x_* is the retention time of the substance (min); *T_y_* is the retention time of n-alkanes whose retention time is less than and closest to the substance (min); *T_z_* is the retention time of n-alkanes whose retention time is greater than and closest to the substance (min). 

Gas chromatography-olfactometry: analyzed by olfactory analyzer. The effluent of gas chromatograph flows into the hydrogen flame ionization detector and olfactory detector respectively at the end of capillary with a 1:1 split ratio. An experienced panel of trained GC-O assessors (*n* = 8) evaluated the effluent of each sample. The results of olfactory evaluation can be recorded by 4 or more assessors at the same time in the same relative olfactory time, and the aromatic compounds can be identified.

### 2.6. Statistical Analysis

The data were analyzed by the SPSS software v22.0 (IBM, Armonk, NY, USA), using a one-way analysis of variance (ANOVA) followed by SNK multiple comparison test or nonparametric tests—Tamhane (T2). Differences with a *p* value of <0.05 were considered statistically significant. A principal component analysis (PCA) of the volatile compounds data for the 30 samples was performed using OriginPro software v2019b (OriginLab, Northampton, MA, USA). The hierarchical cluster analysis of volatile compounds was carried out by MATLAB (MathWorks, Natick, MA, USA). The original variables were pretreated by Z-score method and regard the Euclidean distance (ED) as the standard to obtain the cluster [24]. 

## 3. Results and Discussion

### 3.1. Chemical Composition

Chemical analysis of LTL muscle samples of breeds is shown in Table 2. Compared with Dorper, Tan and Hu lambs had a higher content of intramuscular fat (*p* < 0.05) and a lower content of moisture, crude protein, and ash (*p* < 0.05), indicating that fat-tailed (Tan lamb and Hu lamb in present study) lamb may accumulate more IMF and less crude protein compared to short-tailed lamb (Dorper lamb, lean meat type). Our results echoed Jandasek et al., that IMF content shows a positive correlation with dry matter, and negative correlation with ash content in lambs [25]. Beyond that, our result showed a great improvement in the IMF of Tan lambs compared to grazing ones in previous study [13]. It indicated that the Tan lamb had a potential IMF deposition capacity under the indoor-feeding system.

### 3.2. Fatty Acid Composition

As shown in Table 3, compared to Dorper, Tan lambs had a higher proportion of total saturated fatty acid (SFA) (*p* < 0.05), but breed had no effect on palmitic acid and stearic acid. Total monounsaturated fatty acids (MUFA) and oleic acid had no difference among breeds (Table 4). Oleic acid (C18:1n-9) is one of the volatile compound precursors in lamb meat. Previous study found the lower oleic acid (C18:1n-9) and MUFA level in crossbred lamb muscle than purebred lamb [17]. In another study, different C18:1n-9 contents were found among Suffolk, Soay and Frisland lambs [26]. However, in this study, we did not find any statistical differences among three purebreds in oleic acid and total MUFA. The inconsistent results with the previous studies may be because of the different genotype of lambs, which led to the distinct rumen metabolism and metabolic rate [27], and this will have further influence on the volatile compounds derived from oleic acid.

It has been proven that unsaturated fat in meat varies across different animal species and breeds [19,28]. In our study, Dorper had a higher total polyunsaturated fatty acids (PUFA) proportion than Tan and Hu lambs (*p* < 0.05), which supported the negative correlation between PUFA and IMF [11]. Moreover, we found the Dorper had a higher proportion of EPA (C20:5n-3), DHA (C22:6n-3) and other long-chained PUFAs (Table 5). These PUFAs play a critical role in maintaining membrane structure. So, it is reasonable for Dorper lamb, which is a lean type with less IMF content and higher proportion of long-chained PUFAs.

Linoleic acid (LA, 18:2, n-6) and alpha linolenic acid (ALA, 18:3, n-3) are the most studied PUFA precursors of odor-active compounds [29]. LA and ALA of Dorper were significantly higher than Tan lambs (*p* < 0.05) in the current study. The evidence showed that the increase of LA could help in reducing the mutton-like odor intensity, while enhanced ALA may cause the strong unpleasant odor in lamb meat [30]. Other than that, the oxidation of polyunsaturated fatty acids could affect the volatile odorant [31], and leaner muscle is considered much easier to occur the oxidizing reactions. So, this explained why more volatile compounds derived from linolenic acid were detected in Dorper than Tan and Hu lambs.

n-6 PUFA, n-3 PUFA and P/S ratio of Dorper were significantly higher than Tan and Hu lambs (*p* < 0.05). Consistent with previous studies, we found the negative correlation which occurred between IMF and P/S ratio among breeds [20,32]. It indicated the different fatty acid deposition of short-tailed and fat-tailed lambs. In our study, fat-tailed Hu lambs had a more desirable n−6/n−3 PUFA ratio. But the ratio is still far more than the recommended level 4 or less. From this perspective, under the indoor raising system, Hu lamb may provide a better PUFA constitution.

### 3.3. Amino Acid Composition

Table 6 shows the amino acid profile of LTL of lambs. The amino acids in Dorper were significantly higher than Tan and Hu lambs (*p* < 0.05), which is in response to the result of crude protein. The lean type lambs like Dorper may have had a relative higher requirement for branched-chain amino acids improving the growth of muscle. For example, Leu Iso and Val promoted the translation of mRNA through mTORC1 signal to inhibits protein degradation [33,34]. Hence, protein synthesis was greater in Dorper than the other two breeds.

Sulfur containing amino acids is an important flavor precursor. Hydrogen sulfide produced by the thermal decomposition of cysteine or cystine widely exists in cooked meat and takes part in a series of chemical reactions. We found an enhanced amino acid level in Dorper, including cysteine, methionine, which may contribute to generating hydrogen sulfide and heavy flavor substances [18]. The other important compound phenylthiophenol, which is produced by hydrogen sulfide and phenol, could also affect the mutton smell [35]. These volatile substances were not found in any breeds in this study. It is probably limited by measure approaches. The similar product of cysteine, 2-Methyl-3-furanthiol, failed to be detected either. It is a strong smell compound and the key component to produce the ideal flavor of beef [36,37]. However, too much mercaptan will make mutton produce a peculiar smell [38].

### 3.4. Volatile Compound Composition

**Figure 1:**
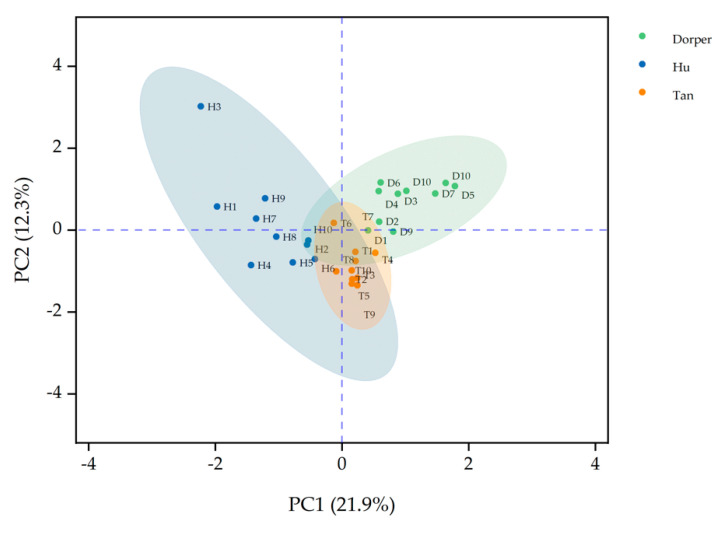
Principal component plot of volatile compounds of lamb meat. PC1: principal component 1 (21.9%), PC2: principal component 2 (12.3%)

**Figure 2:**
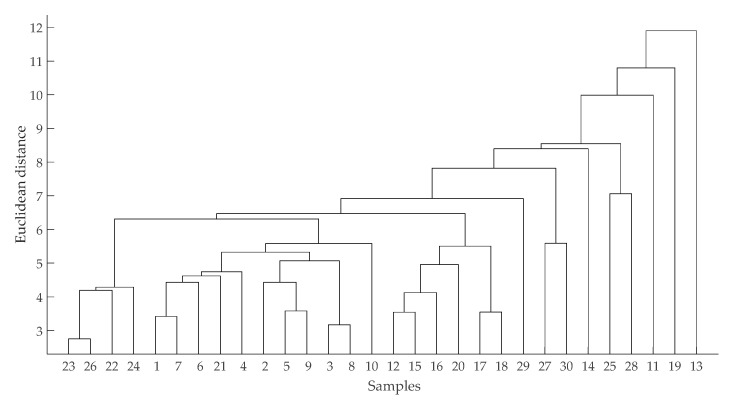
Hierarchical diagram of volatile compounds. Sample 1–10: Tan; Sample 11–20: Hu; Sample 21–30: Dorper.

Figure 1 shows the principal component plot of volatile compounds of three breeds on two major factors (21.9% of PC1, 12.3% of PC2). Obviously, the dots were varying across breeds and overlapped. Figure 2 shows the cluster map of volatile compounds (Figure 2).

An accepted lamb aroma is based on the proper concentration and relative ratios of multiple odor-active compounds. There were 28, 30 and 23 volatile compounds that have been detected in Tan, Dorper and Hu lambs respectively, including aldehydes, alcohols, heterocycles, ketones, and acids (Table 7). No ‘liver’ or ‘taint’ volatile compounds were detected by the trained analyzers. It may because the lambs we tested were all within 1 year old. Most of the odor-active compounds that we identified have been reported in cooked lamb aroma previously [18,38]. The odor profile is determined by the concentration and threshold of volatile compounds [15]. Generally, aldehydes have a relatively lower odor threshold; therefore, aldehydes are considered to have a critical influence on the volatile flavor of lamb [39]. In our study, more than other two breeds, there were 10 kinds of aldehydes have been identified from the volatile compounds in Tan lamb. Moreover, (*E*)-2-hexenal was only found in Tan lamb. It is one of the derivatives produced by alpha linolenic acid (ALA) and was recognized as having a pleasant apple fragrance. Benzaldehyde, another derivative of ALA, was higher in Tan than Dorper lambs (*p* < 0.05). However, the almond- and caramel-like smell of benzaldehyde may have a negative impact on the odor profile. However, Tan had the lowest proportion of ALA, while its derivatives were more numerous than the other two breeds. Furthermore, (*E*)-2-nonenal, (*E*,*E*)-2,4-decadienal and 4-ethylbenzaldehyde are the major substances that are derived from linoleic acid [32]. These aldehydes were only found in Dorper lambs, which also have the highest linoleic acid proportion. Interestingly, Tan had more ALA derivates but less ALA substrate, while Dorper had the most LA derivates with the most LA substrate. Probably, the diverse oxidative reactions of ALA occurred in different breeds, and the variation may be one of the key points to form the different aroma profile. Thus, the intensity of fatty, poultry-like, and rancid smell may be stronger in Dorper lambs.

Aliphatic saturated aldehydes, such as hexanal and octanal, have lower odor thresholds and relative high concentrations in raw and cooked meat; they are the indicators of rancid meat, and might be related to the high proportion of unsaturated fatty acids, like oleic acid [7]. The concentrations of saturated aldehydes are associated with each other and with acid and metallic flavor intensities [40]. In our study, both Tan and Dorper had higher hexanal and octanal concentrations than Hu (*p* < 0.05). Dorper had a higher heptanal concentration than Tan and Hu (*p* < 0.05), but there is no difference among breeds in the concentration of oleic acid. Thus, the results implicate the fat tailed lambs Tan and Hu remain varies in fatty acid oxidation. Similar with ALA derivates, it seems that different reactions happened in breeds. In addition, Hu had the least volatile aldehydes among three breeds, but the highest benzaldehyde content of Hu lambs may strongly affect its overall odor. The different ALA derivates production among breeds may be one of the basic reasons causing the distinction in aroma profiles.

The alcohols have a relatively higher odor threshold, and their contribution to volatile flavor is weaker than that of aldehydes [41]. Even so, alcohol is still a basic factor that influences people’s olfactory perception of the odor profile [42]. In this study, the same nine alcohols were found in three breeds. Although odor threshold of unsaturated alcohols is higher than that of saturated alcohols [43], the 1-octene-3-ol, which presents a mushroom-like smell, has also been identified. We found that 1-octen-3-ol was higher in Dorper than Tan and Hu (*p* < 0.05). On the other side, benzyl alcohol, the aromatic organics with a sweet and flora-like odor, was the highest in Hu (*p* < 0.05). However, pentanol and benzyl alcohol supplied oily or sweet odor and they are not the special aroma in the system of lamb meat. Unlike the results above, we found that Hu had the highest thiophene-3-carboxaldehyde concentration, and Dorper had the lowest one (*p* < 0.05), but thiophene-3-carboxaldehyde failed to be identified by olfactory analyzer, maybe because it has a relatively higher threshold. Among the detected aroma-active heterocyclic compounds, most of the alkyl derivatives are the degradation products by fatty acids. For example, 2-pentylfuran is the oxidative product of linoleic acid [44], and it provides a roast smell. However, 2-pentylfuran was absent in Hu lambs. Besides, most of the sulfur heterocyclic compounds are the derivatives of sulfur-containing amino acids and participate in Maillard reaction, such as 2-acetylpyrrole, which has a nutty and toast-like smell, and is the product of the glutathione glucose reaction system [45]. However, the content of 2-acetylpyrrole has no statistic difference among breeds in our study.

The 3-hydroxy-2-butanone in Hu was significantly higher than Tan and Dorper lambs (*p* < 0.05). The different concentrations of 3-hydroxy-2-butanone affected the aroma profile at varying levels in three breeds. Additionally, the buttery and cream-like smell of 3-hydroxy-2-butanone were easily perceived by olfactory analyzers. We also found that Tan and Hu had higher octanoic acid content than Dorper lambs (*p* < 0.05). However, styrene and hexanoic acid were not found in Hu lambs. In our study, many volatile compounds were absent in Hu lambs, especially aldehydes, which is one of the key factors causing different odor profiles.

## 4. Conclusions

The results of this study demonstrated that Tan and Hu could accumulate more IMF than Dorper, while Dorper provided a larger proportion of PUFA and amino acid content to meet the rapid growth of muscular tissue. Dorper lambs also had an abundance of volatile compounds. However, the specific PUFA derivates of Dorper had a negative impact on the odor profile. However, the typical derivates of Tan influenced the odor profile positively. Hence, the further works should be focused on crossbreed lambs by Dorper and Tan, to enhance lamb production, balance the IMF content and improve meat flavor. 

Additionally, we suggest utilizing more progenies from different sires to provide sufficient genetic coverage, and to be more presentative of the breed characteristics. 

## Figures and Tables

**Table 1:** Composition and nutrient level of diet (Dry-matter basis, %).

Ingredients	Content (%)	Nutrient Level	Content
Pellet		ME ^2^ (MJ/kg)	9.86
Corn	37.33	Crude protein	15.78
Wheat bran	6.67	Crude fiber	14.54
Rapeseed cake	5.33	Neutral detergent fiber	45.39
Soybean meal	5.33	Acid detergent fiber	31.06
Rice bran	4.00	Ether extract	2.34
Corn gluten meal	4.00	Total Calcium	0.50
Nahco_3_	1.33	Total Phosphorus	0.29
Salt	1.33		
Premix ^1^	1.33		
Alfalfa meal	33.33		
Total	100.00		

^1^ Per kg of premix contained VA 850,000 IU, VD_3_ 200,000 IU, VE 2380 IU, Nicotinamide 1930 mg, biotin 2.0 mg, Fe 12 g, Cu 2.5 g, Zn 15 g, Mn 10 g, I 200 mg, Se 50 mg, Co 200 mg. ^2^ The metabolic energy was calculated value.

**Table 2:** Chemical analysis of *Longissimus thoracis et lumborum* (LTL) for Tan, Dorper and Hu.

Trait	Breed			SEM	*p* Value
	Tan (*n* = 10)	Dorper (*n* = 10)	Hu (*n* = 10)		
Moisture (%)	67.24 ^b^	71.71 ^a^	67.92 ^b^	0.492	<0.001
Crude Protein (%)	18.94 ^b^	21.18 ^a^	19.20 ^b^	0.324	<0.001
Intramuscular fat (%)	10.96 ^a^	3.83 ^b^	10.02 ^a^	0.623	0.005
Ash (%)	3.06 ^b^	4.13 ^a^	3.21 ^b^	0.118	<0.001

Different superscripts (a, b) means are significantly different on the same row.

**Table 3:** Effect of breed on saturated fatty acid (%) of LTL.

SFA (%)	Tan (*n* = 10)	Dorper (*n* = 10)	Hu (*n* = 10)	SEM	*p* Value
C10:0	0.10	0.12	0.12	0.004	0.158
C12:0	0.21 ^a^	0.11 ^b^	0.13 ^b^	0.013	0.001
C14:0	3.31 ^a^	2.08 ^b^	2.56 ^b^	0.138	<0.001
C15:0	0.49 ^a^	0.38 ^b^	0.44 ^a^	0.014	0.001
C16:0	24.55	23.93	24.25	0.227	0.554
C17:0	1.49	1.29	1.41	0.059	0.386
C18:0	19.88 ^b^	19.58 ^b^	22.10 ^a^	0.353	0.003
C20:0	0.13 ^b^	0.12 ^b^	0.15 ^a^	0.003	<0.001
C21:0	0.19 ^b^	0.31 ^a^	0.15 ^b^	0.017	<0.001
C22:0	0.04 ^b^	0.05 ^a^	0.03 ^b^	0.002	0.002
C23:0	0.04 ^b^	0.06 ^a^	0.03 ^b^	0.008	<0.001
C24:0	0.03 ^b^	0.04 ^a^	0.02 ^b^	0.002	<0.001
ΣSFA	50.47 ^a^	48.07 ^b^	51.38 ^a^	0.357	<0.001

Different superscripts (a, b) means significant different on the same row.

**Table 4:** Effect of breed on monounsaturated fatty acid (%) of LTL.

MUFA (%)	Tan (*n* = 10)	Dorper (*n* = 10)	Hu (*n* = 10)	SEM	*p* Value
C14:1	0.10 ^a^	0.07 ^b^	0.08 ^b^	0.005	0.025
C16:1	1.55	1.44	1.44	0.037	0.371
C18:1n-9c	40.72	40.37	40.60	0.36	0.645
C20:1	0.13 ^b^	0.14 ^b^	0.15 ^a^	0.002	0.009
C22:1n-9	0.04	0.03	0.04	0.001	0.159
C24:1	0.02 ^b^	0.04 ^a^	0.02 ^b^	0.002	<0.001
ΣMUFA	43.04	42.09	42.32	0.378	0.581

Different superscripts (a, b) means significant different on the same row.

**Table 5:** Effect of breed on polyunsaturated fatty acid (%) of LTL.

PUFA (%)	Tan (*n* = 10)	Dorper (*n* = 10)	Hu (*n* = 10)	SEM	*p* Value
C18:2n-6c	5.09 ^b^	7.17 ^a^	4.94 ^b^	0.313	0.002
C18:3n-3	0.32 ^b^	0.44 ^a^	0.43 ^a^	0.014	<0.001
C20:2	0.02 ^b^	0.04 ^a^	0.02 ^b^	0.002	<0.001
C20:3n-6	0.08 ^b^	0.17 ^a^	0.08 ^b^	0.011	<0.001
C20:4n-6	0.92 ^b^	1.86 ^a^	0.76 ^b^	0.129	<0.001
C20:3n-3	0.01 ^b^	0.03 ^a^	0.01 ^b^	0.002	<0.001
C20:5n-3 (EPA)	0.04 ^b^	0.08 ^a^	0.03 ^b^	0.005	<0.001
C22:6n-3 (DHA)	0.02 ^b^	0.05 ^a^	0.03 ^a^	0.003	<0.001
ΣPUFA	6.49 ^b^	9.84 ^a^	6.30 ^b^	0.468	0.001
P/S	0.13 ^b^	0.20 ^a^	0.12 ^b^	0.010	<0.001
n-6 PUFA	6.08 ^b^	9.20 ^a^	5.78 ^b^	0.446	0.001
n-3 PUFA	0.39 ^c^	0.60 ^a^	0.50 ^b^	0.022	<0.001
n-6/n-3 PUFA	15.28 ^a^	15.22 ^a^	11.55 ^b^	0.402	<0.001

PUFA, polyunsaturated fatty acids. Sum of polyunsaturated FA (ΣPUFA). Polyunsaturated FA: saturated FA (P/S). Different superscripts (a, b, c) means significantly different on the same row.

**Table 6:** Effect of breed on amino acid (%) of LTL.

Amino Acid (%)	Tan (*n* = 10)	Dorper (*n* = 10)	Hu (*n* = 10)	SEM	*p* Value
Aspartic acid	5.51 ^b^	7.00 ^a^	5.53 ^b^	0.159	<0.001
Threonine	2.77 ^b^	3.50 ^a^	2.78 ^b^	0.080	<0.001
Serine	2.32 ^b^	2.95 ^a^	2.57 ^b^	0.069	<0.001
Glutamic acid	9.00 ^b^	11.46 ^a^	9.26 ^b^	0.252	<0.001
Proline	2.32 ^c^	3.11 ^a^	2.69 ^b^	0.084	<0.001
Glycine	2.92 ^b^	3.39 ^a^	3.23 ^ab^	0.072	0.019
Alanine	3.17 ^b^	3.95 ^a^	3.38 ^b^	0.100	0.002
Cysteine	0.84 ^b^	1.01 ^a^	0.83 ^b^	0.024	0.001
Valine	2.77 ^b^	3.87 ^a^	2.98 ^b^	0.105	<0.001
Methionine	2.00 ^b^	2.28 ^a^	1.88 ^b^	0.060	0.015
Isoleucine	2.67 ^b^	3.57 ^a^	2.70 ^b^	0.92	<0.001
Leucine	4.82 ^b^	6.19 ^a^	4.88 ^b^	0.146	<0.001
Tyrosine	2.21 ^b^	2.83 ^a^	2.10 ^b^	0.094	0.001
Phenylalanine	2.34 ^b^	3.04 ^a^	2.37 ^b^	0.072	<0.001
Histidine	1.84 ^b^	2.54 ^a^	1.87 ^b^	0.076	<0.001
Lysine	4.99 ^b^	6.86 ^a^	5.34 ^b^	0.177	<0.001
Arginine	3.72 ^b^	4.81 ^a^	3.97 ^b^	0.109	<0.001
Tryptophan	0.66 ^b^	0.85 ^a^	0.64 ^b^	0.022	<0.001

Different superscripts (a, b, c) means significantly different on the same row.

**Table 7:** Effect of breed on major Odor-Active Volatile Compounds of Cooked LTL.

Volatile Compounds		Retention Index	Relative Content/%			SEM	*p* Value	Odorant Descriptors
			Tan (*n* = 10)	Dorper (*n* = 10)	Hu (*n* = 10)			
Aldehydes	Hexanal	1081	18.29 ^a^	18.22 ^a^	10.46 ^b^	1.38	0.021	Herbal, grassy
	Heptanal	1183	14.24 ^b^	37.00 ^a^	13.28 ^b^	3.24	0.002	Oily, stock
	(*E*)-2-hexenal	1266	1.309					Apple, fruit
	Octanal	1290	13.01 ^a^	16.66 ^a^	4.76 ^b^	1.28	<0.001	Citrus, floral
	Nonanal	1394	14.97	12.30	9.21	1.15	0.093	Fatty, sweet
	(*E*)-2-octenal	1408	2.88 ^b^	6.89 ^a^	0.95 ^c^	0.54	<0.001	Nutty, meaty
	Benzaldehyde	1495	9.85 ^b^	5.24 ^c^	25.07 ^a^	2.39	<0.001	Almond, caramel
	(*E*)-2-nonenal	1538		1.263				Fatty, rancid
	(*E*)-2-decenaldehyde	1621	1.39	1.08	3.73	1.38	0.688	Chicken fat, orange
	Phenylacetaldehyde	1639	0.82 ^b^	1.30 ^a^	0.56 ^b^	0.11	0.008	Honey, sweet
	4-ethylbenzaldehyde	1707		0.311				bitter almond
	(*E*)-2-undecenal	1754	0.30 ^b^	1.56 ^a^		0.20	<0.001	Wax, fatty
	(*E*,*E*)-2,4-decadienal	1811		1.52				Chicken fat, poultry
Alcohols	Pentanol	1255	1.49 ^b^	4.28 ^c^	2.53 ^b^	0.33	<0.001	Oily
	Hexanol	1360	1.28	1.44	1.40	0.10	0.825	Pine, fruit
	1-octene-3-ol	1453	5.66 ^b^	9.90 ^a^	5.28 ^b^	0.76	0.023	Mushroom, lavender
	Heptanol	1461	1.61	1.66	2.25	0.23	0.462	Grassy, fresh
	2-ethylhexanol	1491	3.08	1.08	2.21	0.38	0.129	Rosy, sweet
	Linalool	1537	0.38	0.06	0.26	0.06	0.105	Floral, tea
	Octanol	1564	2.07	2.04	2.44	0.20	0.676	Burnt
	(*E*)-2-octenol	1620	0.55	1.28	2.86	0.47	0.120	Fatty
	Benzyl alcohol	1806	0.78 ^b^	0.62 ^b^	2.95 ^a^	0.34	0.001	Sweet, flora
Heterocyclic compounds	Furfuryl alcohol	1199	0.56	0.65	0.76	0.06	0.452	Bitter, burnt
	2-pentylfuran	1224	12.11	11.47		1.87	0.871	Roast, buttery
	Thiophene-3-carboxaldehyde	1800	0.17 ^c^	0.61 ^b^	0.92 ^a^	0.08	<0.001	-
	2-acetylpyrrole	1974	0.56	0.57	2.45	0.42	0.125	Nutty, toast
Ketones	3-hydroxy-2-butanone	1287	3.44 ^b^	3.70 ^b^	17.85 ^a^	2.36	0.005	Buttery, cream
	2-undecanone	1543	0.28	0.27	0.40	0.04	0.442	Orange, grassy
Acids	Hexanoic acid	1803	0.95 ^a^	0.58 ^b^		0.07	0.006	Sweat, mutton
	Octanoic acid	2083	0.14 ^a^	0.03 ^b^	0.12 ^a^	0.01	<0.001	Cheese, mutton
Alkenes	Styrene	1251	12.85	9.34		1.10	0.113	Sweet, flora

Different superscripts (a, b, c) means are significantly different on the same row.
